# The relationship of examinees’ individual characteristics and perceived acceptability of smart device-based testing to test scores on the practice test of the Korea Emergency Medicine Technician Licensing Examination

**DOI:** 10.3352/jeehp.2018.15.33

**Published:** 2018-12-27

**Authors:** Eun Young Lim, Mi Kyoung Yim, Sun Huh

**Affiliations:** 1 Division of Educational Evaluation, Korea Institute for Curriculum and Evaluation, Jincheon, Korea; 2 Research and Development Division, Korea Health Personnel Licensing Examination Institute, Seoul, Korea; 3 Department of Parasitology and Institute of Medical Education, College of Medicine, Hallym University, Chuncheon, Korea; The Catholic University of Korea, Korea

**Keywords:** Emergency medical technicians, Licensure, Linear models, Personal satisfaction, Students, Republic of Korea

## Abstract

**Purpose:**

Smart device-based testing (SBT) is being introduced into the Republic of Korea’s high-stakes examination system, starting with the Korean Emergency Medicine Technician Licensing Examination (KEMTLE) in December 2017. In order to minimize the effects of variation in examinees’ environment on test scores, this study aimed to identify any associations of variables related to examinees’ individual characteristics and their perceived acceptability of SBT with their SBT practice test scores.

**Methods:**

Of the 569 candidate students who took the KEMTLE on September 12, 2015, 560 responded to a survey questionnaire on the acceptability of SBT after the examination. The questionnaire addressed 8 individual characteristics and contained 2 satisfaction, 9 convenience, and 9 preference items. A comparative analysis according to individual variables was performed. Furthermore, a generalized linear model (GLM) analysis was conducted to identify the effects of individual characteristics and perceived acceptability of SBT on test scores.

**Results:**

Among those who preferred SBT over paper-and-pencil testing, test scores were higher for male participants (mean± standard deviation [SD], 4.36± 0.72) than for female participants (mean± SD, 4.21± 0.73). According to the GLM, no variables evaluated— including gender and experience with computer-based testing, SBT, or using a tablet PC—showed a statistically significant relationship with the total score, scores on multimedia items, or scores on text items.

**Conclusion:**

Individual characteristics and perceived acceptability of SBT did not affect the SBT practice test scores of emergency medicine technician students in Korea. It should be possible to adopt SBT for the KEMTLE without interference from the variables examined in this study.

## Introduction

Computer-based testing (CBT) has been successfully used for high-stakes medical health licensing examinations in the United States, Canada, and Taiwan. In the Republic of Korea, 24 medical health licensing examinations are managed by the Korea Health Personnel Licensing Examination Institute (KHPLEI). The KHPLEI decided to introduce CBT for the Korean Emergency Medical Technician Licensing Examination (KEMTLE), which is one of the 24 medical health licensing exams managed by the KHPLEI, starting in late 2017 [1,2]. The KEMTLE is the first professional licensing examination that will use SBT in Korea.

The KHPLEI began to administer CBT practice tests in 2014, and decided to introduce smart device-based testing (SBT), which involves the use of a tablet PC instead of a desktop PC. A tablet PC was chosen to avoid placing limitations on testing locations and the number of examinees. If a desktop PC is used for the exam, specifically equipped test centers would be needed, and the number of desktop PCs at the test center would limit the number of examinees. In contrast, using a tablet PC for the exam increases flexibility in the testing locations, and enables the administration of as many exams as the KHPLEI provides tablet PCs for. Therefore, in this report, we use the term SBT instead of CBT. Based on the results of the practice test scores and the questionnaire on examinees’ perceived acceptability of SBT, it may be possible to identify individual characteristics and acceptability-related variables that affect test scores. If such variables are found, we would need to make an effort to minimize their effects in order to achieve comparability between SBT scores and conventional test scores.

In a recent study on SBT in Korea, satisfaction with, convenience of, and preference for SBT compared to paper-and-pencil testing were sufficient to determine that administering SBT was worthwhile [3]. In a focus group interview after CBT at a medical school in Korea, CBT was reported to be good for student learning because it strengthened the clinical context [4]. In another study, experience with computers and anxiety about computers did not affect the CBT test scores of health professions students [5]. In medical school in the United States, content familiarity was found to be related to differences in performance, but not gender, competitiveness, or familiarity with computers [6]. Although some evidence suggests that individual characteristics might affect CBT test scores, more extensive research is needed on the impacts of those characteristics and the perceived acceptability of SBT on SBT test scores. Therefore, we aimed to determine whether individual characteristics and perceived acceptability affected the test scores of examinees on the KEMTLE practice test using SBT. Specifically, we investigated whether individual characteristics affected the perceived acceptability of SBT and whether individual characteristics and perceived acceptability affected the test scores. The acceptability variables consisted of 3 subcategories: satisfaction with, convenience of, and preference for SBT. The null hypotheses of this study were as follows: first, variables relating to individual characteristics would not affect perceived acceptability; and second, variables relating to individual characteristics and perceived acceptability would not affect examinees’ test scores.

## Methods

### Ethics approval

Students participated in the survey after providing written informed consent. This study was approved by the Institutional Review Board of Hallym University (HIRB-2015-092).

### Study design

The study had an observational design based on test results and a questionnaire survey. A generalized linear model (GLM) analysis was conducted to evaluate the effects of individual characteristics and perceived acceptability of SBT on test scores.

### Setting

**Fig. 1.**
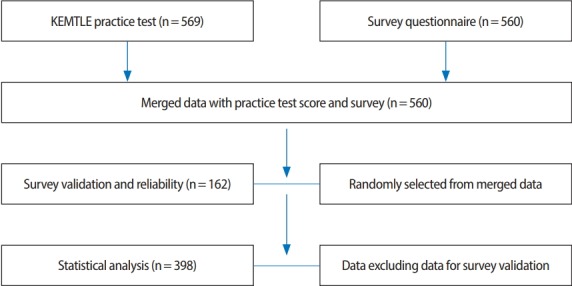
Diagram of the study process. KEMTLE, Korea Emergency Medicine Technician Licensing Examination.

The SBT KEMTLE practice test and questionnaire were administered to 569 candidate students (examinees) at the same sitting on September 12, 2015 in Daejon, Korea. A smart device (a 10-inch tablet PC) was distributed to each examinee, and they marked their responses on the screen of the device. The test items consisted of 50 multimedia items and 80 text items. They were given 120 minutes to complete the examination. All items contained 5 options with 1 best answer. All 569 examinees who were present took the examination; and 560 students responded to the questionnaire on the acceptability of SBT after the examination. The original questionnaires consisted of 8 items regarding individual characteristics, as well as 2 satisfaction, 13 convenience, and 16 preference items (Supplement 1), but based on the results of exploratory factor analysis, 9 convenience and 9 preference items were selected for this study. Items were scored on a 5-point Likert scales (1, strongly disagree; 2, disagree; 3, neutral; 4, agree; 5, strongly disagree). The questionnaire was also administered on the tablet PC. The exam and questionnaire were not internet-based; instead, stand-alone tablet-based testing was used. After the examination and survey, the data in the tablet PCs were moved to a separate location and the responses were transferred to a server. The collected data comprised the test scores of the examinees (Supplement 1) and their responses to the survey questionnaire. Fig. 1 presents a diagram of the study process.

### Participants

A total of 569 examinees were included from the 41 emergency medicine technician schools in Korea, who were arbitrarily selected to be administered the practice test and questionnaire on the perceived acceptability of SBT. They were in their final year of study (i.e., third-year students from 3-year programs or fourth-year students from 4-year programs). The total annual enrollment in the 41 schools was 1,400 based on a national regulation; therefore, the 569 participants corresponded to 40.6% of the target population. The characteristics of the participants are presented in greater detail in Table 1. Of the 569 subjects who took the examination, 560 participated in the questionnaire survey. The validity test was conducted using responses from 162 students, and responses from the other 398 students were used for the null test.

### Variables

The variables related to individual characteristics and perceived acceptability of SBT are listed in Tables 1-4. The examinees’ test scores were considered to be the outcome. The variables for individual characteristics were treated as dichotomous values. The variables for acceptability were on a 5-point Likert scale. Test scores were a continuous variable.

### Data sources/measurement

The source of all variables was response data from the survey questionnaire. The measurement methods were exploratory factor analysis for validity, the Cronbach alpha for reliability of the survey items on perceived acceptability of SBT, the t-test for the relationships of individual variables with perceived acceptability, and a GLM for the effects of variables related to individual characteristics and perceived acceptability on test scores.

### Bias

There was no noteworthy source of bias in data collection or analysis. Nine of the 569 examinees did not respond to the acceptability questionnaire after SBT; this was low enough to have a negligible influence on the analysis.

### Study size

The sample size (N= 569) corresponded to 40.6% of the total target student population, and examinees were drawn from 100% of the 41 emergency medicine technician schools; therefore, the sample size in this study was sufficient for the statistical analysis to be representative of the student population.

### Quantitative variables

All variables were quantitative. They were subjected to a parametric analysis.

### Statistical methods

Three procedures were conducted to test 2 null hypotheses. First, the survey questionnaire on the acceptability of SBT was validated and its reliability was confirmed; second, t-test analyses were performed to evaluate relationships between individual variables and perceived acceptability of SBT; and third, a GLM analysis was conducted to evaluate the effects of individual characteristics and perceived acceptability of SBT on test scores.

To confirm the validity of the questionnaire on the acceptability of SBT, exploratory factor analysis was conducted with the principal axis for the factor extraction method and varimax for factor rotation with 162 examinees. A total of 560 subjects were arbitrarily divided into 2 groups for survey validation (N= 162) and analysis using the t-test and GLM (N= 398). Reliability was assessed using the Cronbach alpha.

To test the null hypotheses, t-test analyses were performed with the results of the questionnaire on the acceptability of SBT and test scores according to the background variables of gender, age, type of university, and experience with CAT, SBT, and use of a tablet PC. Test scores on the KEMTLE were used as the dependent variable. The KEMTLE used for the practice test was composed of 130 items, including multimedia items and text items.

To determine the effect of individual characteristics and perceived acceptability of SBT on test scores, 3 different GLM models were analyzed using 3 different sets of test scores as dependent variables, with the same independent variables that were analyzed using the ttest. More specifically, GLM analyses were conducted of test scores on all 130 items (total scores), test scores on the 50 multimedia items, and test scores on the 80 text items. For this study, 14 variables were available: 6 categorical variables related to individual background characteristics, and 5 factors from the questionnaire regarding perceived acceptability of SBT and the 3 different types of test scores. The factors relating to perceived acceptability of SBT and the test scores were continuous variables. For the GLM analyses, examinees’ characteristics, which were used as independent variables, were selected based on the t-test results. Furthermore, 3 composites derived from the questionnaire on the acceptability of SBT were employed as independent variables (satisfaction with SBT, convenience of each of two SBT features, item solving, and the interface), as well as 2 factors related to preferences for SBT compared to paperand-pencil testing and compared to CBT. SAS ver. 9.4 (SAS Institute Inc., Cary, NC, USA.) was used for the analysis.

## Results

### Descriptive data of participants

Table 1 shows the number of examinees who responded to the survey questionnaire based on their background, subdivided according whether their responses were used for survey validation or the t-test and GLM analysis.

### Outcome

The outcomes of this study were 6 variables related to individual characteristics, their perceived acceptability of SBT, and 3 sets of test scores from the practice examination (Supplement 1).

### Validity and reliability of the acceptability questionnaire

Tables 2 and 3 present the results of exploratory factor analysis of the scale for the convenience of SBT features and the scale for preferences for SBT, respectively.

In addition to these 2 scales, overall satisfaction with using SBT was included in the SBT evaluation survey. The survey was composed of 3 scales: a scale for satisfaction with SBT (2 items), the scale for the convenience of SBT features (9 items), and the scale for preferences for SBT (9 items). The scale for the convenience of SBT features was composed of 2 factors (convenience related to item-solving, and convenience related to the user interface). The scale for preferences for SBT was also composed of 2 factors (preference for SBT compared to CBT and preference for SBT compared to paperand-pencil testing). Table 4 shows the description, the number of items, and the Cronbach alpha coefficient of each scale in the SBT evaluation survey. The range of reliability of scales and factors in each scale for the evaluation survey was 0.836 (convenience of SBT features relative to computer-based test) to 0.920 (preference for SBT). All scales and the factors in each scale showed strong internal consistency and a high level of reliability.

### Descriptive statistics of 8 variables

Table 5 presents the descriptive statistics of the 8 variables related to test scores and the perceived acceptability of SBT.

### Effects of individual background characteristics on the SBT evaluation survey and test scores

Tables 6-10 show the mean values and standard deviations of the results of the SBT evaluation survey according to background variables and their t-test results. The mean results of the evaluation survey by each background category were higher than 3.81 (the mean score for satisfaction with SBT among examinees who had no experience of using a tablet PC) and examinees had high values of satisfaction with SBT, convenience of SBT features, and preference for SBT. The t-test analyses did not yield many statistically significant results. The mean differences in preferences for SBT (t= 2.132, degrees of freedom [df]= 396) and preference for SBT compared to paper-and-pencil testing (t= 2.076, df= 396) by gender were statistically significant at the level of 0.05. No other statistically significant results were found. The mean score for preferences for SBT among male participants (mean± standard deviation [SD], 4.23± 0.63) was higher than among female participants (mean± SD, 4.08± 0.66). Furthermore, the mean score for preference for SBT compared to paper-and-pencil testing among male participants (mean±SD, 4.36± 0.72) was higher than among female participants (mean± SD, 4.21± 0.73). The gender difference in preferences for SBT might have reflected gender differences in adaptability and favorable attitudes to using new information technology. Thus, for the GLM analyses, we needed to confirm whether preferences for SBT or gender affected test scores.

Tables 11-16 show the means and standard deviations of test scores by background variables and their t-test results. No statistically significant relationships were found for any background variables. The mean differences between categories of each background variable were small; for example, the difference between the total mean scores of males (mean± SD, 78.13± 14.008) and those of females (mean± SD, 77.02± 14.45) was 1.11.

### Effects of independent variables on test scores

Based on the t-test results, gender was the independent variable that showed a significant association with preference for SBT (t=2.132, df= 396) and preference for SBT compared to paper-andpencil testing (t= 2.076, df= 396). Gender was included in the GLM analysis, and age and type of university were excluded. Experiences of CBT, SBT, and using a tablet PC were included in the models because they were closely related to the test methods. Table 17 shows an analysis of variance (ANOVA) summary table for total scores, scores on multimedia items, and scores on text items. The R^2^ values of the ANOVA model of the dependent variables were 0.024, 0.023, and 0.024, and the independent variables explained about 2% of the variation in each dependent variable. Table 18 shows the regression coefficients and the values for statistical significance; no variables showed a statistically significant relationship with test scores. Furthermore, the η^2^ values of independent variables were small, indicating that the effect sizes of the independent variables were small.

## Discussion

### Key results

Our main results are as follows. First, the variables related to individual characteristics did not affect the perceived acceptability of SBT by emergency medicine technician students in Korea who took the KEMTLE practice examination, except for effects of gender on preferences for SBT in general and preference for SBT compared to paper-and-pencil testing. Second, the variables related to individual characteristics, satisfaction with SBT, and convenience of SBT did not affect the test scores on the KEMTLE practice examination. The null hypothesis was not rejected; therefore, the adoption of SBT for the KEMTLE should not be a problem for emergency medicine technician students in Korea.

### Limitations

A limitation of this study is that a comparability study between paper-and-pencil tests and SBT was not conducted. However, doing so would be difficult because multimedia items cannot be included in a paper-and-pencil test, and the scores of SBT including multimedia items cannot be compared directly with paper-and-pencil test scores.

### Interpretation

Proficiency or experience with the test device may be a major discriminating factor that could affect the validity of the test. Our results showed no difference in the perceptions of SBT according to experience with SBT or CBT and experience of use of smart devices. We also looked into whether test scores varied according to perceptions and experience with SBT or CBT or use of smart devices. We did not find any significant differences in test scores depending on experience with CBT or SBT. The average SBT exam scores of examinees with experience of CBT and those with no experience were 79.00 and 76.74, respectively. The scores of examinees with and without SBT experience were 78.21 and 77.51, respectively. The average test score of those with experience using smart devices was 77.6; while that of those who were not current users was 71.3. The Experience using a smart device seems to have influenced the test score. However, very few students did not have experience using smart devices (18; 4.5% of all participants), so the results for experience with use of smart devices should be interpreted with care.

### Generalizability

The number of subjects eligible for this study was 1,400 from 41 emergency medicine technician schools. Of these students, 569 were selected for SBT and 560 (98.4%) responded to the questionnaire survey; therefore, the sample of this study can reasonably be considered representative of the total population of the emergency medicine technician students.

### Conclusion

Two null hypotheses of this study were accepted. SBT can be adopted for the KEMTLE without difficulties arising from the variables examined in this study.

## Figures and Tables

**Table 1.** Background of the 560 emergency medicine technician students who took the exam in 2015 in Korea and responded to the questionnaire

Background	Frequency (%)	
	Survey validation (N=162)	t-test and generalized linear model analysis (N=398)
Gender		
Male	91 (56.2)	227 (57.0)
Female	71 (43.8)	171 (43.0)
Age (yr)		
20–24	127 (78.4)	312 (78.4)
≥ 25	35 (21.6)	86 (21.6)
Program length (yr)		
3	98 (60.5)	222 (55.8)
4	64 (39.5)	176 (44.2)
Experience taking a computer-based test		
Yes	74 (45.7)	161 (40.5)
No	88 (54.3)	237 (59.5)
Experience taking a smart device-based test		
Yes	20 (12.3)	80 (20.1)
No	142 (87.7)	318 (79.9)
Experience using a smart device		
Yes	160 (98.8)	380 (95.5)
No	2 (1.2)	18 (4.5)

**Table 2.** Results of exploratory factor analysis of the scale for the convenience of smart device-based testing features from 162 emergency medicine technician students in 2015 in Korea

Convenience of smart device-based testing	Factor	
	1	2
It was convenient to check the items that were not solved before submitting the answers.	0.755	0.270
The functions for selecting the correct answer and changing the selection were convenient.	0.736	0.322
The functions for seeing the previous item, the next item, and the list of all items was convenient.	0.728	0.282
It was convenient to see one item on one screen.	0.604	0.292
The indication of the time remaining on the test was more convenient than announcements of the remaining time.	0.516	0.267
The screen user interface was appropriate.	0.261	0.849
The loading time was adequate for video playing.	0.300	0.705
Zooming in and out of the figure and replaying of video were appropriate.	0.329	0.669
The font was good.	0.367	0.580

**Table 3.** Results of exploratory factor analysis of the scale for preferences for smart device-based testing features from 162 emergency medicine technician students in 2015 in Korea

Preference for smart device-based testing	Factor	
	1	2
Tablet PC-based testing is an improved system over existing desktop PC-based testing.	0.754	0.291
Finger touch input to the tablet PC was more convenient than mouse-click input to a desktop PC.	0.752	0.363
Cheating may be less likely when using a tablet PC than when using a desktop PC.	0.745	0.275
The test device was simple and convenient, allowing me to focus more on the test.	0.737	0.475
There was no noise or heat from the test device, so that the test environment was comfortable.	0.718	0.215
There was less eye strain with the tablet PC than with the desktop PC.	0.693	0.349
The psychological burden (tension) decreased because there was no answer-marking procedure on the OMR card.	0.236	0.772
The font size was large and clear, so that it was convenient for me to solve the problems.	0.332	0.771
The lack of an answer-marking procedure on the OMR card helped me allocate time for solving items.	0.389	0.724

OMR, optical mark reader.

**Table 4.** The content, number of items, and Cronbach alpha coefficient of each scale in the questionnaire on the perceived acceptability of SBT administered to 162 emergency medicine technician students in 2015 in Korea (N=162)

Scale (description)	Content factor	No. of items	Mean±standard deviation	Cronbach alpha
Satisfaction with SBT		2	4.20 ± 0.88	0.875
Convenience of SBT (degree of convenience of SBT features)	All	9	4.30 ± 0.58	0.885
	1) Related to solving items	5	4.40 ± 0.62	0.836
	2) Related to user-interface	4	4.19 ± 0.66	0.849
Preference for SBT (preference for SBT compared to paper-and-pencil testing and computer-based testing)	All	9	4.20 ± 0.66	0.920
	1) Compared to computer-based testing	6	4.13 ± 0.73	0.905
	2) Compared to paper-and-pencil testing	3	4.35 ± 0.70	0.898

SBT, smart device-based testing.

**Table 5.** Descriptive statistics of 8 variables related to test scores and perceived acceptability of SBT from 398 emergency medical technician students in Korea in 2015 (N=398)

Continuous variable	Min	Max	Mean ± standard deviation
Test scores			
Total score (no. of items = 115)	33	115	77.65 ± 14.23
Score on multimedia items (no. of items = 50)	15	43	29.32 ± 5.44
Scores on text items (no. of items = 80)	18	75	48.33 ± 9.77
Satisfaction with SBT	1.00	5.00	4.01 ± 0.98
Convenience of SBT features			
1) Solving items	2.20	5.00	4.35 ± 0.66
2) Interface	2.00	5.00	4.13 ± 0.71
Preference for SBT			
1) Compared to paper-and-pencil testing	1.83	5.00	4.10 ± 0.74
2) Compared to computer-based testing	2.00	5.00	4.30 ± 0.72

SBT, smart device-based testing.

**Table 6.** Means, standard deviations, and t-test results of the SBT evaluation survey by gender

Acceptability of SBT	Male (N = 227)	Female (N = 171)	t-value
Satisfaction of using SBT	4.01 ± 1.04	4.00 ± 0.90	0.166
Convenience of SBT features			
All	4.27 ± 0.64	4.23 ± 0.61	0.505
1) Item-solving	4.37 ± 0.67	4.32 ± 0.66	0.676
2) Interface	4.14 ± 0.74	4.12 ± 0.68	0.210
Preferences for SBT			
All	4.23 ± 0.67	4.08 ± 0.69	2.132^*^
1) Compared to computer-based testing	4.16 ± 0.72	4.01 ± 0.75	1.942
2) Compared to paper-and-pencil testing	4.36 ± 0.72	4.21 ± 0.73	2.076^*^

Values are presented as mean±standard deviation. SBT, smart device-based testing.

* P<0.05.

**Table 7.** Means, standard deviations, and t-test results of the SBT evaluation survey by age

Acceptability of SBT	Age (yr)		t-value
	20–24 (N = 312)	≥ 25 (N = 86)	
Satisfaction of using SBT	4.00 ± 0.96	4.01 ± 1.07	-0.057
Convenience of SBT features			
All	4.26 ± 0.63	4.21 ± 0.61	0.685
1) Item-solving	4.36 ± 0.66	4.32 ± 0.67	0.438
2) Interface	4.15 ± 0.71	4.07 ± 0.73	0.841
Preferences for SBT			
All	4.13 ± 0.71	4.27 ± 0.59	-1.774
1) Compared to computer-based testing	4.07 ± 0.76	4.19 ± 0.66	-1.405
2) Compared to paper-and-pencil testing	4.27 ± 0.74	4.41 ± 0.65	-1.813

Values are presented as mean±standard deviation. SBT, smart device-based testing.

**Table 8.** Means, standard deviations, and t-test results of the SBT evaluation survey by program length

Acceptability of SBT	Program length		t-test
	3 yr (N = 222)	4 yr (N = 176)	
Satisfaction with SBT	4.02 ± 0.96	3.99 ± 1.00	0.371
Convenience of SBT features			
All	4.28 ± 0.61	4.22 ± 0.64	1.012
1) Item-solving	4.38 ± 0.64	4.31 ± 0.69	1.122
2) Interface	4.15 ± 0.73	4.1 ± 0.70	0.690
Preferences for SBT			
All	4.20 ± 0.68	4.12 ± 0.69	1.137
1) Compared to computer-based testing	4.13 ± 0.75	4.05 ± 0.72	0.990
2) Compared to paper-and-pencil testing	4.34 ± 0.69	4.25 ± 0.76	1.202

Values are presented as mean±standard deviation. SBT, smart device-based testing.

**Table 9.** Means, standard deviations, and t-test results of the SBT evaluation survey by experience with CBT

Acceptability of SBT	Experience taking CBT		t-value
	Yes (N = 161)	No (N = 237)	
Satisfaction with SBT	4.07 ± 0.94	3.96 ± 1.01	1.041
Convenience of SBT features			
All	4.23 ± 0.65	4.27 ± 0.61	-0.643
1) Item-solving	4.34 ± 0.69	4.36 ± 0.65	-0.299
2) Interface	4.09 ± 0.74	4.16 ± 0.69	-0.920
Preferences for SBT			
All	4.22 ± 0.70	4.13 ± 0.67	1.293
1) Compared to computer-based testing	4.16 ± 0.76	4.05 ± 0.72	1.461
2) Compared to paper-and-pencil testing	4.33 ± 0.72	4.28 ± 0.73	0.685

Values are presented as mean±standard deviation. SBT, smart device-based testing; CBT, computer-based testing.

**Table 10.** Means, standard deviations, and t-test results of the SBT evaluation survey by experience using a smart device

Acceptability of SBT	Experience using a smart device		t-value
	Yes (N=80)	No (N=318)	
Satisfaction with SBT	4.02 ± 0.98	3.81 ± 0.96	0.889
Convenience of SBT features			
All	4.25 ± 0.63	4.23 ± 0.60	0.160
1) Solving-items	4.35 ± 0.67	4.37 ± 0.62	-0.117
2) Interface	4.13 ± 0.72	4.06 ± 0.70	0.452
Preferences for SBT			
All	4.17 ± 0.68	4.00 ± 0.72	1.036
1) Compared to computer-based testing	4.10 ± 0.74	3.94 ± 0.75	0.887
2) Compared to paper-and-pencil testing	4.31 ± 0.72	4.11 ± 0.81	1.126

Values are presented as mean±standard deviation. SBT, smart device-based testing.

**Table 11.** Means, standard deviations, and t-test results of test scores by gender

Scores	Gender		t-value
	Male (N = 227)	Female (N = 171)	
Total score	78.13 ± 14.08	77.02 ± 14.45	0.766
Score on multimedia items	29.27 ± 5.42	29.39 ± 5.48	-0.223
Score on text items	48.86 ± 9.70	47.63 ± 9.85	1.241

Values are presented as mean±standard deviation.

**Table 12.** Means, standard deviations, and t-test results of test scores by age

Scores	Age (yr)		t-value
	20–24 (N = 312)	≥ 25 (N = 86)	
Total score	77.81 ± 14.36	77.08 ± 13.83	0.420
Score on multimedia items	29.39 ± 5.53	29.06 ± 5.13	0.507
Score on text items	48.42 ± 9.79	48.02 ± 9.75	0.330

Values are presented as mean±standard deviation.

**Table 13.** Means, standard deviations, and t-test results of test scores by program length

Scores	Program length		t-value
	3 yr (N=222)	4 yr (N=176)	
Total score	77.38 ± 14.26	77.99 ± 14.23	-0.425
Score on multimedia items	29.19 ± 5.43	29.48 ± 5.46	-0.526
Score on text items	48.19 ± 9.83	48.51 ± 9.72	-0.326

Values are presented as mean±standard deviation.

**Table 14.** Means, standard deviations, and t-test results of test scores by experience with computer-based testing

Scores	Experience with computer-based testing		t-value
	Yes (N=161)	No (N=237)	
Total score	79.00 ± 12.54	76.74 ± 15.23	1.617
Score on multimedia items	29.67 ± 4.80	29.08 ± 5.83	1.095
Score on text items	49.33 ± 8.86	47.65 ± 10.31	1.732

Values are presented as mean±standard deviation.

**Table 15.** Means, standard deviations, and t-test results of test scores by experience with smart device-based testing

Scores	Experience with smart device-based testing		t-value
	Yes (N=80)	No (N=318)	
Total score	78.21 ± 14.68	77.51 ± 14.14	0.393
Score on multimedia items	29.39 ± 5.17	29.31 ± 5.51	0.121
Score on text items	48.83 ± 10.51	48.21 ± 9.59	0.505

Values are presented as mean±standard deviation.

**Table 16.** Means, standard deviations, and t-test results of test scores by experience using a smart device

Scores	Experience using a smart device		t-value
	Yes (N=380)	No (N=18)	
Total score	77.87 ± 14.38	73.06 ± 9.88	1.404
Score on multimedia items	29.41	27.39	1.545
Score on text items	48.46 ± 9.87	45.67 ± 7.25	1.185

Values are presented as mean±standard deviation.

**Table 17.** Analysis of variance summary table for total scores, scores on multimedia items, and scores on text items

Dependent variable	Source	Degrees of freedom	Sum of squares	Mean square	F-value	Pr ( > F-value)
Total score (R^2^ = 0.024)	Model	10	2,401,945	240,194.5	1,188.15	< 0.0001
	Error	388	78,437.14	202.158		
	Total	398	2,480,382			
Score on multimedia items (R^2^ = 0.023)	Model	10	2,401,945	240,194.5	1,188.15	< 0.0001
	Error	388	78,437.14	202.158		
	Total	398	2,480,382			
Score on text items (R^2^ = 0.024)	Model	10	342,453.8	34,245.38	1,157.4	< 0.0001
	Error	388	11,480.22	29.5882		
	Total	398	353,934			

**Table 18.** Regression coefficients and statistical significance of the generalized linear model of test scores

Test score	Independent variable	Parameter estimates				
		B	Standard error	t-value	P-value	η^2^
Total score	Intercept	72.68	6.06	11.99	< 0.0001	0.3436
	Male	1.57	1.48	1.06	0.289	0.0029
	Experience taking a computer-based test: yes	2.67	1.57	1.70	0.090	0.0074
	Experience taking a smart device-based test: yes	-1.03	1.94	-0.53	0.597	0.0007
	Experience using a smart device: yes	4.95	3.48	1.42	0.156	0.0052
	Satisfaction with SBT	-0.79	0.86	-0.92	0.357	0.0022
	Convenience of SBT features: solving problems	0.18	1.61	0.11	0.912	0
	Convenience of SBT features: interface	2.63	1.54	1.71	0.089	0.0075
	Preference for SBT compared to computer-based testing	-0.01	1.48	-0.01	0.993	0
	Preference for SBT compared to paper-and-pencil testing	-2.31	1.61	-1.43	0.152	0.0053
Score on multimedia items	Intercept	27.10	2.32	11.69	< 0.0001	0.3287
	Male	0.09	0.57	0.17	0.8689	0.0001
	Experience taking a computer-based test: yes	0.78	0.60	1.29	0.1979	0.0043
	Experience taking a smart device-based test: yes	-0.42	0.74	-0.57	0.5686	0.0008
	Experience using a smart device: yes	2.16	1.33	1.62	0.106	0.0067
	Satisfaction with SBT	-0.27	0.33	-0.83	0.4086	0.0018
	Convenience of SBT features: solving problems	0.56	0.61	0.91	0.364	0.0021
	Convenience of SBT features: interface	0.89	0.59	1.51	0.1329	0.0058
	Preference for SBT compared to computer-based testing	-0.18	0.57	-0.32	0.7501	0.0003
	Preference for SBT compared to paper-and-pencil testing	-1.02	0.62	-1.65	0.098	0.007
Score on text items	Intercept	45.58	4.16	10.95	< 0.0001	0.306
	Male	1.48	1.01	1.45	0.1466	0.0054
	Experience taking a computer-based test: yes	1.90	1.08	1.75	0.0801	0.0079
	Experience taking a smart device-based test: yes	-0.60	1.33	-0.45	0.6503	0.0005
	Experience using a smart device: yes	2.79	2.39	1.17	0.2438	0.0035
	Satisfaction with SBT	-0.52	0.59	-0.88	0.3787	0.002
	Convenience of SBT features: solving problems	-0.38	1.10	-0.34	0.7304	0.0003
	Convenience of SBT features: interface	1.74	1.06	1.65	0.1001	0.007
	Preference for SBT compared to computer-based testing	0.17	1.02	0.17	0.8687	0.0001
	Preference for SBT compared to paper-and-pencil testing	-1.29	1.10	-1.17	0.2436	0.0035

SBT, smart device-based testing.

## Supplementary material

Supplement 1. Data files are available from https://doi.org/10.7910/DVN/UUIC8V.

Supplement 2. Audio recording of the abstract.
